# Efficacy of tofacitinib in patients with rheumatoid arthritis stratified by background methotrexate dose group

**DOI:** 10.1007/s10067-016-3436-1

**Published:** 2016-10-12

**Authors:** R Fleischmann, PJ Mease, S Schwartzman, L-J Hwang, K Soma, CA Connell, L Takiya, E Bananis

**Affiliations:** 1 Metroplex Clinical Research Center and University of Texas Southwestern Medical Center, Dallas, TX USA; 2 Swedish Medical Center and University of Washington School of Medicine, Seattle, WA USA; 3 Hospital for Special Surgery, New York, NY USA; 4 Pfizer Inc, New York, NY USA; 5 Pfizer Inc, Groton, CT USA; 6 Pfizer Inc, Collegeville, PA USA

**Keywords:** Disease activity, Janus kinase, Methotrexate, Radiographic progression, Rheumatoid arthritis, Tofacitinib

## Abstract

Tofacitinib is an oral Janus kinase inhibitor for the treatment of rheumatoid arthritis (RA). This post hoc analysis investigated the effect of methotrexate (MTX) dose on the efficacy of tofacitinib in patients with RA. ORAL Scan (NCT00847613) was a 2-year, randomized, Phase 3 trial evaluating tofacitinib in MTX-inadequate responder (IR) patients with RA. Patients received tofacitinib 5 or 10 mg twice daily (BID), or placebo, with low (≤12.5 mg/week), moderate (>12.5 to <17.5 mg/week), or high (≥17.5 mg/week) stable background MTX. Efficacy endpoints (at months 3 and 6) included American College of Rheumatology (ACR) 20/50/70 response rates, and mean change from baseline in Clinical Disease Activity Index (CDAI), Disease Activity Score in 28 joints (DAS28)–4(erythrocyte sedimentation rate [ESR]), Health Assessment Questionnaire-Disability Index (HAQ-DI), and modified Total Sharp score. 797 patients were treated with tofacitinib 5 mg BID (*N* = 321), tofacitinib 10 mg BID (*N* = 316), or placebo (*N* = 160); 242, 333, and 222 patients received low, moderate, and high MTX doses, respectively. At months 3 and 6, ACR20/50/70 response rates were greater for both tofacitinib doses vs placebo across all MTX doses. At month 3, mean changes from baseline in CDAI and HAQ-DI were significantly greater for both tofacitinib doses vs placebo, irrespective of MTX category; improvements were maintained at month 6. Both tofacitinib doses demonstrated improvements in DAS28–4(ESR), and less structural progression vs placebo, across MTX doses at month 6. Tofacitinib plus MTX showed greater clinical and radiographic efficacy than placebo in MTX-IR patients with RA, regardless of MTX dose.

**Electronic supplementary material:**

The online version of this article (doi:10.1007/s10067-016-3436-1) contains supplementary material, which is available to authorized users.

## Introduction

Current guidelines advise that treatment of rheumatoid arthritis (RA) should be initiated with conventional synthetic disease-modifying antirheumatic drugs (csDMARDs), such as methotrexate (MTX). For patients with inadequate response (IR) to csDMARDs, options include biologic DMARDs (bDMARDs), such as tumor necrosis factor inhibitors (TNFi), or targeted synthetic small-molecule DMARDs (tsDMARDs), often given in combination with csDMARDs [1, 2].

Early intervention with csDMARDs, usually MTX, has been shown to be beneficial in achieving a good clinical response in a proportion of patients, with resulting modification of the course of the disease [1, 3, 4]. However, not all patients achieve remission with MTX. There is extensive evidence to suggest that concomitant use of a bDMARD with MTX is beneficial in achieving treatment goals [5–12]. However, it is not clear whether there is a minimum or maximum dose of MTX that, when given in combination with bDMARDs, can affect clinical outcomes. Recently, a study has shown that increasing doses of MTX administered concomitantly with adalimumab broadly associated with an increasing proportion of patients achieving clinical efficacy endpoints [13, 14]. A recent study in Japan has also shown that adalimumab plus ≥10 mg MTX consistently resulted in better improvement in disease activity score in 28 joints (DAS28) and more patients with DAS28-defined remission compared with adalimumab plus MTX <10 mg [15].

Tofacitinib is an oral Janus kinase inhibitor for the treatment of RA. The efficacy and safety of tofacitinib 5 and 10 mg twice daily (BID), as monotherapy or in combination with csDMARDs, have been demonstrated in Phase 2 and Phase 3 clinical trials and in long-term extension studies [16–27]. The tofacitinib clinical development program included both MTX-naïve patients and those with an IR to treatment with DMARDs, including csDMARDs (primarily MTX) and TNFi.

ORAL Scan (NCT00847613) was a Phase 3 clinical trial that investigated tofacitinib 5 and 10 mg BID vs placebo, all with stable background MTX, in MTX-IR patients with RA [24]. In ORAL Scan, tofacitinib plus MTX improved the signs and symptoms of RA, clinical disease activity, and reduced the progression of structural damage compared with placebo plus MTX at month 6. The structural damage results were statistically significant with tofacitinib 10 mg BID (*p* < 0.05), but not with tofacitinib 5 mg BID (*p* = 0.0792) [24].

This post hoc analysis of data from ORAL Scan investigated whether there was a difference in the benefit of tofacitinib when given with oral MTX at different dose ranges.

## Materials and methods

### Study design, patients, and MTX dose categories

ORAL Scan (NCT00847613) was a 2-year, multinational, randomized, double-blind, parallel-group, placebo-controlled Phase 3 clinical trial conducted in accordance with the Declaration of Helsinki and International Conference on Harmonisation Guidelines for Good Clinical Practice. The Institutional Review Board at each study center approved the protocol and all patients provided written informed consent.

Details of the study design and patient population have been reported previously [24]. Briefly, eligible patients were aged ≥18 years with active RA despite receiving MTX at a stable dose of 15–25 mg/week for ≥6 weeks (MTX doses <15 mg/week were permitted if there were safety issues at higher doses, but dose adjustments were not permitted during the study). Patients were randomized 4:4:1:1 to receive tofacitinib 5 mg BID, tofacitinib 10 mg BID, placebo advanced to tofacitinib 5 mg BID, or placebo advanced to tofacitinib 10 mg BID, all in combination with MTX. Placebo patients advanced to active treatment at month 3 if they did not meet a pre-defined response of ≥20 % improvement in swollen and tender joint counts. All remaining placebo patients mandatorily advanced to their assigned tofacitinib dose at month 6 regardless of clinical response.

In this post hoc analysis, patients were divided into three categories according to their baseline mean weekly MTX dose: low (≤12.5 mg/week), moderate (>12.5 to <17.5 mg/week), and high (≥17.5 mg/week). MTX doses were stable throughout the study as per protocol.

### Efficacy assessments

Signs and symptoms and clinical disease activity were assessed at months 3 and 6 utilizing American College of Rheumatology 20, 50, and 70 % response rates (ACR20/50/70), least squares mean (LSM) change from baseline in Clinical Disease Activity Index (CDAI) score, and the proportion of patients achieving low disease activity (CDAI score ≤10) and remission (CDAI score ≤2.8). LSM change from baseline in DAS28–4(erythrocyte sedimentation rate [ESR]) was assessed at month 6.

Functional status was assessed by LSM change from baseline in the Health Assessment Questionnaire-Disability Index (HAQ-DI) score, including the proportion of patients achieving HAQ-DI <0.5.

Radiographic progression was assessed via LSM change from baseline in van der Heijde modified Total Sharp Score (mTSS), and the proportion of patients with no radiographic progression, defined as ≤0.5-unit increase from baseline in mTSS at month 6.

### Statistical analyses

In this exploratory, post hoc analysis, the primary analysis population was the Full Analysis Set, which included all randomized patients who received ≥1 dose of study medication and had ≥1 post-baseline measurement. To handle missing data, no imputation was applied to descriptive statistics of patient demographics and baseline characteristics. For binary efficacy endpoints, non-responder imputation (NRI) was applied. In particular, patients who withdrew from a study for any reason before month 6, or patients who advanced treatment to active tofacitinib after month 3, had their values on or after withdrawing or advancing treatment set to “non-responder” in the efficacy analyses. Longitudinal continuous variables were analyzed using a mixed-effect repeated measure model with no imputation for missing data. The fixed effects of treatment, visit, and treatment-by-visit interaction were included, with subject as a random effect.

This was a post hoc analysis, and no adjustment was made for multiple testing. For the efficacy analyses, 95 % confidence intervals (CIs) for each tofacitinib group vs placebo were calculated in each MTX dose category; a CI that did not contain 0 was taken to indicate that the difference between tofacitinib and placebo was significant. Logistic regression analyses for binary variables and linear regression analyses for continuous variables were used to evaluate the effect of various baseline factors (CDAI, DAS28–4[ESR], HAQ-DI, body mass index [BMI], glucocorticoid [GC] use, MTX dose, swollen joints, and tender joints) on efficacy responses. A multivariate model was developed that incorporated variables from a univariate model with *p* values <0.10 and removed factors using stepwise, backward, and forward methods until only significant factors remained (*p* < 0.05).

## Results

### Patients

A total of 797 patients were randomized to receive tofacitinib 5 mg BID (*N* = 321), tofacitinib 10 mg BID (*N* = 316), or placebo (*N* = 160), all in combination with background MTX; patients continued to receive concomitant MTX at the dose they were receiving at study start. The number of patients in the low, moderate, and high MTX dose categories was 242 (30 %), 333 (42 %), and 222 (28 %), respectively (Table 1). The mean weekly MTX dose in the three categories was 9.4, 15.0, and 20.9 mg/week, respectively (Table 1).

**Table 1:** Patient demographics and baseline characteristics

	Low MTX (≤12.5 mg/week) (*N* = 242)			Moderate MTX (>12.5 to <17.5 mg/week) (*N* = 333)			High MTX (≥17.5 mg/week) (*N* = 222)		
	Placebo (*n* = 47)	Tofacitinib 5 mg BID (*n* = 102)	Tofacitinib 10 mg BID (*n* = 93)	Placebo (*n* = 73)	Tofacitinib 5 mg BID (*n* = 131)	Tofacitinib 10 mg BID (*n* = 129)	Placebo (*n* = 40)	Tofacitinib 5 mg BID (*n* = 88)	Tofacitinib 10 mg BID (*n* = 94)
Mean weekly dose of MTX, mg	9.1	9.6	9.4	15.0	15.0	15.0	21.2	21.0	20.6
Caucasian, %	42.6	33.3	26.9	42.5	50.4	49.6	52.5	59.1	58.5
Female, %	80.9	84.3	91.4	94.5	89.3	86.8	75.0	75.0	79.8
Mean age (SD), years	55.1 (11.8)	53.3 (12.1)	52.7 (11.2)	51.6 (11.0)	53.7 (11.4)	51.8 (11.1)	51.6 (12.3)	54.1 (11.3)	51.5 (12.2)
Mean duration of RA (SD), years	9.7 (7.4)	9.5 (8.4)	8.4 (8.2)	10.0 (10.7)	8.9 (7.8)	9.3 (8.3)	8.5 (8.1)	8.2 (6.9)	9.2 (8.5)
Mean BMI (SD), kg/m^2^	24.5 (6.6)	24.3 (5.3)	23.9 (4.9)	26.1 (6.1)	26.4 (5.7)	26.2 (6.4)	28.6 (6.2)	29.2 (8.1)	27.3 (6.0)
Concomitant GC use, %	51.1	70.6	67.7	68.5	69.5	65.9	80.0	76.1	74.5
Prior TNFi therapy, %	21.3	22.5	17.2	5.5	18.3	17.8	2.5	17.0	11.7
Prior non-TNFi therapy, %	4.3	4.9	4.3	4.1	4.6	3.9	0.0	5.7	6.4
LSM mTSS (SE)	37.1 (8.2)	42.7 (5.4)	38.5 (5.7)	36.4 (5.7)	27.9 (4.2)	35.2 (4.3)	30.4 (7.9)	25.7 (5.4)	40.7 (5.2)
Mean swollen joint count (SD)	14.0 (9.1)	13.8 (6.8)	13.4 (5.7)	12.4 (6.4)	13.9 (8.6)	14.4 (8.0)	17.6 (9.7)	14.8 (9.1)	15.6 (8.8)
Mean tender joint count (SD)	20.0 (11.8)	21.3 (12.3)	19.9 (11.8)	22.1 (12.3)	24.6 (13.8)	24.2 (14.9)	27.8 (14.9)	26.8 (15.7)	24.3 (16.0)
Mean HAQ-DI (SD)	1.38 (0.69)	1.38 (0.71)	1.45 (0.57)	1.33 (0.66)	1.41 (0.69)	1.36 (0.66)	1.22 (0.71)	1.45 (0.64)	1.39 (0.75)
Mean DAS28–4(ESR) (SD)	6.3 (1.0)	6.2 (0.9)	6.3 (0.8)	6.2 (1.0)	6.5 (0.9)	6.2 (0.9)	6.5 (1.1)	6.3 (1.1)	6.2 (1.2)
Mean CDAI (SD)	32.6 (11.5)	33.3 (10.9)	33.9 (10.4)	32.8 (11.7)	36.5 (11.6)	34.8 (12.0)	38.8 (14.0)	36.6 (12.3)	36.0 (13.8)

*BID* twice daily, *BMI* body mass index, *CDAI* Clinical Disease Activity Index, *DAS28–4(ESR)* disease activity score in 28 joints, erythrocyte sedimentation rate, *GC* glucocorticoid, *HAQ-DI* Health Assessment Questionnaire-Disability Index, *LSM* least squares mean, *mTSS* modified Total Sharp/van der Heijde Score, *MTX* methotrexate, *RA* rheumatoid arthritis, *SD* standard deviation, *SE* standard error, *TNFi* tumor necrosis factor inhibitor

Baseline demographics and disease characteristics were generally similar across the MTX dose categories. BMI, proportion of Caucasian patients, GC use, swollen and tender joint counts, and CDAI scores tended to be higher among patients in the high MTX dose category, and proportion of patients with prior TNFi therapy, which tended to be higher in the low MTX dose category (Table 1).

### Efficacy

#### Clinical and functional outcomes

**Fig. 1:**
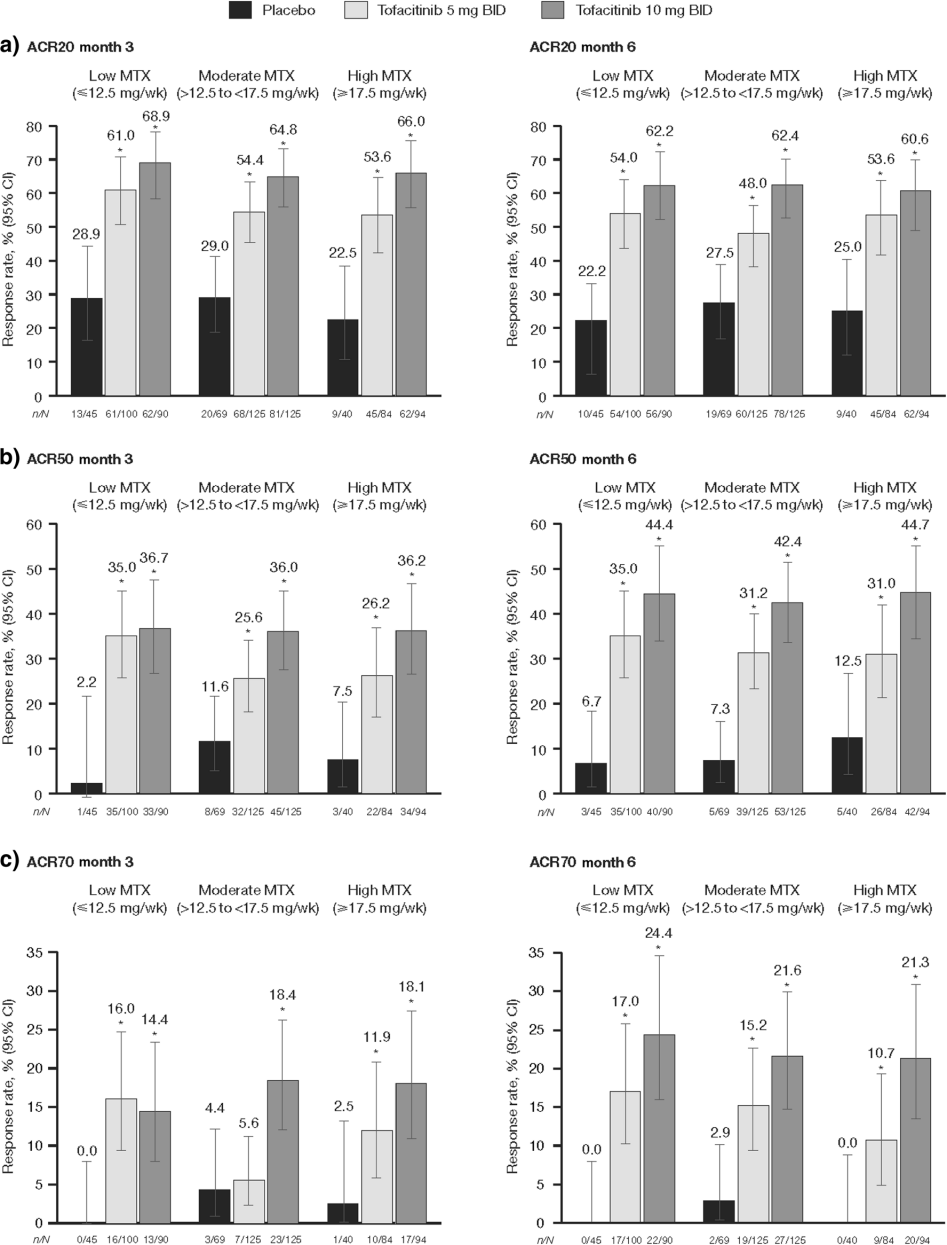
Proportion of patients achieving **a** ACR20, **b** ACR50, and **c** ACR70 at month 3 and month 6. *p < 0.05 vs placebo plus MTX. *ACR* American College of Rheumatology, *BID* twice daily, *CI* confidence interval, *MTX* methotrexate, *n* number of patients responding, *N* number of patients assessed, *wk* week

The proportion of patients achieving ACR20/50/70 response rates was significantly greater for those receiving tofacitinib 5 and 10 mg BID vs placebo, regardless of MTX dose level, at both month 3 and month 6. The only exception was ACR70 for tofacitinib 5 mg BID at month 3 in the moderate MTX dose group, which was numerically greater than placebo (Fig. 1).

**Fig. 2:**
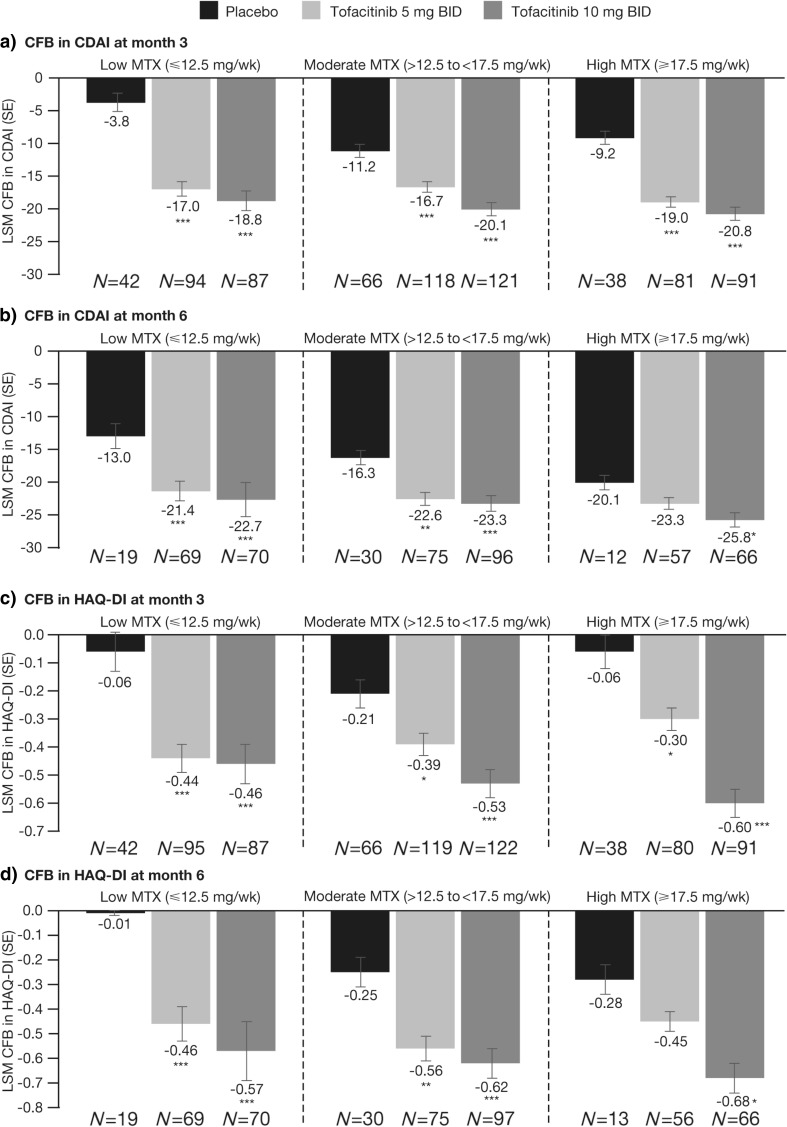
Change from baseline in **a** CDAI at month 3, **b** CDAI at month 6, **c** HAQ-DI at month 3, and **d** HAQ-DI at month 6. *p < 0.05, **p < 0.001, ***p < 0.0001 vs placebo; Full Analysis Set. *BID* twice daily, *CDAI* Clinical Disease Activity Index, *CFB* change from baseline, *HAQ-DI* Health Assessment Questionnaire-Disability Index, *LSM* least squares mean, *MTX* methotrexate, *N* number of patients assessed, *SE* standard error, *wk* week

Tofacitinib 5 and 10 mg BID-treated patients achieved significantly greater reductions from baseline in CDAI scores at month 3, compared with placebo, irrespective of MTX dose (Fig. 2a). Other than the tofacitinib 5 mg BID vs placebo comparison in the high MTX dose group, significant improvements in CDAI scores (Fig. 2b) and DAS28–4(ESR) scores (Table 2) were maintained at month 6 in all three MTX dose categories.

**Table 2:** Selected efficacy endpoints at month 6 by MTX dose category

	Low MTX (≤12.5 mg/week) (*N* = 242)			Moderate MTX (>12.5 to <17.5 mg/week) (*N* = 333)			High MTX (≥17.5 mg/week) (*N* = 222)		
	Placebo (*n* = 47)	Tofacitinib 5 mg BID (*n* = 102)	Tofacitinib 10 mg BID (*n* = 93)	Placebo (*n* = 73)	Tofacitinib 5 mg BID (*n* = 131)	Tofacitinib 10 mg BID (*n* = 129)	Placebo (*n* = 40)	Tofacitinib 5 mg BID (*n* = 88)	Tofacitinib 10 mg BID (*n* = 94)
*Disease activity* ^a^									
CDAI ≤10, % (95 % CI)	8.9 (2.5, 21.2)	42.0* (32.2, 52.3)	52.2* (41.4, 62.9)	13.0 (6.1, 23.3)	32.0* (23.9, 40.9)	41.1* (32.4, 50.3)	12.5 (4.2, 26.8)	35.7* (25.6, 46.9)	39.4* (29.4, 50.0)
CDAI ≤2.8, % (95 % CI)	2.2 (0.1, 11.8)	9.0 (4.2, 16.4)	13.3* (7.1, 22.1)	1.5 (0.0, 7.8)	8.8* (4.5, 15.2)	13.7* (8.2, 21.0)	0 (0.0, 8.8)	9.5* (4.2, 17.9)	16.0* (9.2, 25.0)
LSM CFB in DAS28–4(ESR) (SE)	−1.00 (0.26)	−2.15* (0.13)	−2.48* (0.14)	−1.33 (0.18)	−2.28* (0.11)	−2.39* (0.10)	−2.07 (0.27)	-2.14 (0.13)	-2.67* (0.13)
*Functional status* ^a^									
HAQ-DI <0.5, % (95 % CI)	0 (0.0, 7.9)	24.0* (16.0, 33.6)	24.4* (16.0, 34.6)	5.8 (1.6, 14.2)	17.6* (11.4, 25.4)	29.8* (22.0, 38.7)	10.0 (2.8, 23.7)	19.1 (11.3, 29.1)	33.0* (23.6, 43.4)

*BID* twice daily, *CDAI* Clinical Disease Activity Index, *CFB* change from baseline, *CI* confidence interval, *DAS28–4(ESR)* disease activity score in 28 joints, erythrocyte sedimentation rate, *HAQ-DI* Health Assessment Questionnaire-Disability Index, *LSM* least squares mean, *MTX* methotrexate, *SE* standard error

*p < 0.05 vs placebo plus MTX; ^a^Non-responder imputation; Full Analysis Set

At month 6, significantly higher proportions of patients treated with both doses of tofacitinib achieved CDAI low disease activity (CDAI score ≤10) and remission (CDAI score ≤2.8) compared with the placebo group, regardless of background MTX dose, with the exception of the tofacitinib 5 mg group with low-dose MTX for CDAI remission (Table 2).

Improvements from baseline in HAQ-DI were significantly greater for tofacitinib 5 and 10 mg BID vs placebo in all MTX dose groups at months 3 and 6, other than tofacitinib 5 mg BID in the high-dose MTX group at month 6 (Fig. 2c, d). At month 6, significantly higher proportions of patients treated with tofacitinib 5 and 10 mg BID vs placebo in all MTX dose groups achieved HAQ-DI <0.5, except for tofacitinib 5 mg BID in the high-dose MTX group (Table 2).

#### Radiographic progression

**Fig. 3:**
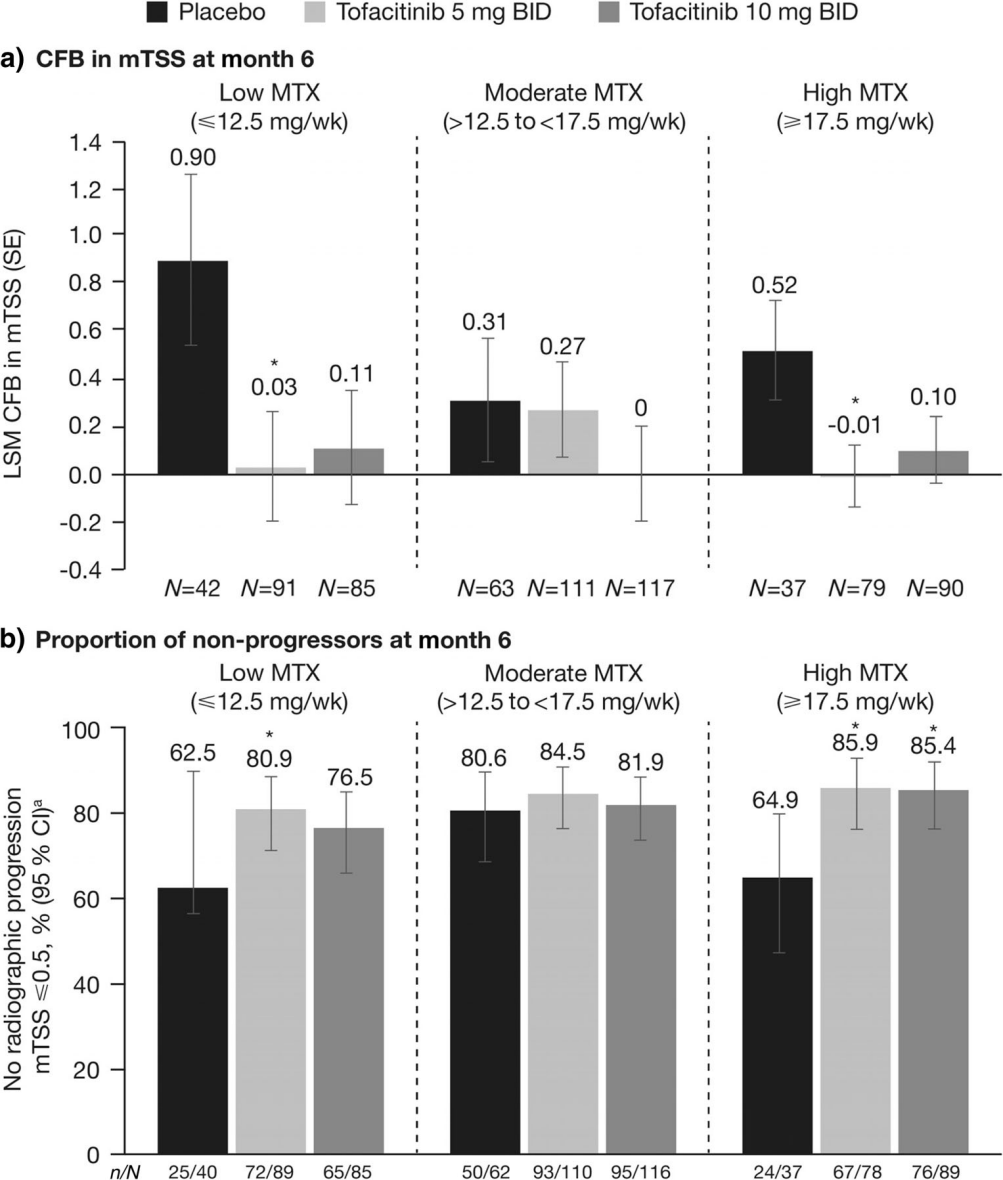
Radiographic progression at month 6 by MTX dose category: **a** LSM change from baseline in mTSS and **b** proportion of patients with no radiographic progression (mTSS ≤0.5). *p < 0.05 vs placebo; ^a^Non-responder imputation; Full Analysis Set. *BID* twice daily, *CFB* change from baseline, *CI* confidence interval, *LSM* least squares mean, *mTSS* modified Total Sharp/van der Heijde Score, *MTX* methotrexate, *SE* standard error, *wk* week

No clear relationship between radiographic progression (as measured by LSM change from baseline in mTSS scores and the proportion of patients with no radiographic progression) and MTX dose was observed at month 6 with tofacitinib 5 or 10 mg BID (Fig. 3a, b). Generally, less radiographic progression was observed with both tofacitinib 5 and 10 mg BID plus MTX compared with placebo plus MTX (Fig. 3a, b). In the placebo-treated patients, less radiographic progression was demonstrated in the moderate and high MTX dose groups compared with the low MTX dose group (Fig. 3a).

#### Efficacy analyses by MTX dose and baseline variables

The univariate and multivariate regression analyses performed to assess the effect of baseline variables on efficacy outcomes showed no significant effect of BMI, GC use, or MTX dose on disease activity assessments with either tofacitinib 5 or 10 mg BID (Supplementary Table 1 in Online Resource 1).

## Discussion

It has been shown that the concomitant use of a bDMARD with MTX can be clinically beneficial in MTX-IR patients; what has not been demonstrated conclusively is whether there is a minimum dose of MTX that, when given in combination with bDMARDs, affects clinical outcomes. In a previous post hoc analysis of data from the tofacitinib clinical RA program, broadly similar efficacy was seen in studies with tofacitinib administered as monotherapy and tofacitinib given in combination with MTX [28]. Whereas the earlier analysis used data from four different clinical studies, this post hoc analysis of data from the Phase 3 ORAL Scan study was performed to establish whether the efficacy of tofacitinib 5 mg BID or 10 mg BID is affected by the dose of concomitant MTX within a single study. The MTX-IR population for this post hoc analysis was similar to MTX-IR patients with RA who are candidates for tofacitinib or bDMARDs in clinical practice. The finding of whether there is a dose-dependent effect of concomitant MTX on clinical efficacy with tofacitinib is therefore clinically relevant.

Analysis of data from the ORAL Scan study revealed that both tofacitinib 5 and 10 mg BID were more effective in improving clinical activity and functional status in RA patients compared with placebo, regardless of the background MTX dose. The proportion of patients achieving ACR20/50/70 response rates, CDAI LDA ≤10, CDAI remission ≤2.8, and HAQ-DI <0.5 from baseline were greater for both tofacitinib doses compared with placebo, regardless of MTX dose level. Improvements from baseline in HAQ-DI, CDAI, and DAS28–4(ESR) were greater for both tofacitinib doses plus MTX compared with placebo plus MTX, regardless of MTX dose level.

Generally, less radiographic progression was observed at month 6 with both tofacitinib doses plus MTX compared with placebo plus MTX; the inhibition of radiographic progression at month 6 with either dose of tofacitinib did not appear to be MTX dose-dependent. In this post hoc analysis, tofacitinib 5 mg BID plus MTX showed significantly lower radiographic progression vs placebo plus MTX at month 6 in the low and high MTX dose groups, but a significant difference was not observed in the overall analysis [24]. In general, as expected, at month 6, higher doses of MTX in the placebo-treated patients with background MTX achieved improved clinical response and improved functional status with less radiographic progression than the lowest dose of MTX.

Concomitant use of MTX with all currently approved bDMARDs has consistently been associated with better efficacy than bDMARD monotherapy, but studies examining the effect of MTX dose on the efficacy of bDMARDs have reported differing findings. In a trial of MTX- and bDMARD-naïve patients treated with adalimumab 40 mg every other week in combination with MTX (2.5, 5, 10, or 20 mg per week), a trend of increased efficacy with increasing MTX dose was noted; however, the efficacy of adalimumab plus 10 or 20 mg of MTX weekly appeared equivalent [13]. Similar results were reported in the Dutch RhEumatoid Arthritis Monitoring (DREAM) registry [14]. In this report, there did not appear to be a difference in efficacy of adalimumab, etanercept, or infliximab in patients receiving 10, 15, 20, or 25 mg/week concomitant MTX. In the MUSICA trial, in which patients were treated with adalimumab 40 mg every other week in combination with low- or high-dose MTX (7.5 or 20 mg per week), small differences in terms of efficacy were observed between the two MTX groups but higher responses and some significant differences were reported with high-dose MTX [29]. In another study, a sub-analysis of pooled Phase 3 data of certolizumab pegol revealed no difference in clinical effect with different MTX doses [30].

It has been postulated that the mechanism for the increased efficacy of bDMARDs, especially monoclonal antibodies, with concomitant MTX vs bDMARD monotherapy is the impact of MTX on serum levels of bDMARDs. A study on the concentration-effect curve of adalimumab demonstrated that concomitant administration of MTX was associated with significantly higher trough levels of adalimumab compared with adalimumab monotherapy; the median level with concomitant MTX use was within the range of adalimumab concentrations thought necessary for clinical efficacy [31]. One mechanism of clearance of adalimumab from serum is related to the production of antidrug antibodies (ADAb) [31], with patients producing higher amounts of ADAb having reduced serum concentrations of functional adalimumab [32]. MTX, an immunomodulator, has been shown to reduce the immunogenicity of adalimumab in a dose-dependent manner [33]. An investigation of the co-administration of MTX 15–25 mg/week with tofacitinib in patients with RA reported no clinically significant effect on the pharmacokinetic profile of either drug [34].

Limitations of this post hoc analysis include low patient numbers in some treatment groups. The analysis could not determine definitively whether there is a differential effect of tofacitinib plus MTX depending on MTX dose. There were also some differences between groups in baseline characteristics, although these did not appear to affect efficacy assessments and interpretation. In the present analysis, patients had to have active disease despite treatment with MTX for inclusion in the study and therefore had limited response to MTX, irrespective of the dose. However, the majority of patients responded when tofacitinib was added to MTX. To determine the effect of MTX dose on the efficacy of tofacitinib, a study that includes csDMARD-, bDMARD-, and ts-DMARD-naïve patients assigned to varying doses of MTX plus concomitant tofacitinib or MTX monotherapy is required, similar to the study conducted by Burmester and colleagues for adalimumab [13]. Such a study could show unequivocally whether there is a dose response of MTX on tofacitinib efficacy, together with information on any differences in safety with increasing MTX dose.

In conclusion, this post hoc analysis confirms the main findings from ORAL Scan. Tofacitinib plus MTX generally showed greater clinical and radiographic efficacy than placebo plus MTX in MTX-IR patients with RA. The findings reported here suggest that in this population, the effect of tofacitinib is irrespective of background MTX dose, and that concomitant high-dose MTX may not be needed for tofacitinib efficacy.

## Electronic supplementary material

ESM 1 (DOC 95 kb)
