# Supplementary material for: Efficacy of tofacitinib in patients with rheumatoid arthritis stratified by background methotrexate dose group
Source: Clin Rheumatol. 2016 Oct 12;36(1):15–24. doi: 10.1007/s10067-016-3436-1 (PMC5216063; doi:10.1007/s10067-016-3436-1)
Supplement: Supplementary file 1 — (DOC 95 kb) [file 10067_2016_3436_MOESM1_ESM.doc]

**Online Resource 1**

***Clinical Rheumatology***

**Efficacy of tofacitinib in patients with rheumatoid arthritis stratified by background methotrexate dose group**

R Fleischmann,^1^ PJ Mease,^2^ S Schwartzman,^3^ L-J Hwang,^4^ K Soma,^5^ CA Connell,^5^ L Takiya,^6^ E Bananis^6^

*^1^Metroplex Clinical Research Center and University of Texas Southwestern Medical Center, Dallas, TX, USA; ^2^Swedish Medical Center and University of Washington School of Medicine, Seattle, WA, USA; ^3^Hospital for Special Surgery, New York, NY, USA; ^4^Pfizer Inc, New York, NY, USA; ^5^Pfizer Inc, Groton, CT, USA*; *^6^Pfizer Inc, Collegeville, PA, USA*

**Corresponding author:**Eustratios Bananis
Pfizer Inc
500 Arcola Road, Collegeville, PA 19426, USA
Telephone: +1 484 865 6688
Email: [stratis.bananis@pfizer.com](mailto:stratis.bananis@pfizer.com)

**Supplementary Table 1.** Univariate and multivariate regression analyses of baseline factors affecting efficacy responses

| **Efficacy outcome at Month 6** | **Significant baseline factors (p<0.05)** | | **Non-significant factors in**  **multivariate analysis** |
| --- | --- | --- | --- |
|  | **Univariate analysis** | **Multivariate analysis^a^** |  |
| **All patients** | | | |
| ACR20 | BMI, SJ^b^ | BMI, SJ^b^ | GC, MTX, TJ |
| CFB in HAQ-DI | HAQ-DI^c^ | HAQ-DI^c^ | BMI, GC, MTX |
| CFB in DAS28-4(ESR) | DAS28-4(ESR)^c^ | DAS28-4(ESR)^c^ | BMI, GC, MTX |
| CDAI ≤10 | CDAI^d^ | CDAI^d^ | BMI, GC, MTX |
| CFB in CDAI | CDAI^c^ | CDAI^c^ | BMI, GC, MTX |
| **All tofacitinib-treated patients** | | | |
| ACR20 | SJ^b^ | SJ^b^ | BMI, GC, MTX, TJ |
| CFB in HAQ-DI | HAQ-DI^c^ | HAQ-DI^c^ | BMI, GC, MTX |
| CFB in DAS28-4(ESR) | DAS28-4(ESR)^c^ | DAS28-4(ESR)^c^ | BMI, GC, MTX |
| CDAI ≤10 | CDAI,^d^ moderate vs low MTX | CDAI^d^ | BMI, GC, MTX |
| CFB in CDAI | CDAI^c^ | CDAI^c^ | BMI, GC, MTX |

^a^Multivariate model incorporated variables from the univariate model with p values <0.10; ^b^higher=better probability; ^c^higher=larger improvement; ^d^lower=better probability.

ACR20, American College of Rheumatology 20% response; BMI, body mass index; CDAI, Clinical Disease Activity Index; CFB, change from baseline; DAS28-4(ESR), disease activity score in 28 joints, erythrocyte sedimentation rate; GC, glucocorticoid; HAQ-DI, Health Assessment Questionnaire-Disability Index; MTX, methotrexate; SJ, swollen joints; TJ, tender joints.
